# Increased rates of protein evolution and asymmetric deceleration after the whole-genome duplication in yeasts

**DOI:** 10.1186/s12862-017-0895-1

**Published:** 2017-02-06

**Authors:** Diana Ascencio, Soledad Ochoa, Luis Delaye, Alexander DeLuna

**Affiliations:** 1 Unidad de Genómica Avanzada (Langebio), Centro de Investigación y de Estudios Avanzados del IPN, Irapuato, Guanajuato Mexico; 2 Departamento de Ingeniería Genética, Centro de Investigación y de Estudios Avanzados del IPN, Irapuato, Guanajuato Mexico

**Keywords:** Gene duplication, Whole-genome duplication, Protein-evolution rate, Purifying selection, Asymmetric evolution, Phylogenetic analysis by maximum likelihood, Biological function, *Saccharomyces cerevisiae*

## Abstract

**Background:**

Whole-genome duplication (WGD) events have shaped the genomes of eukaryotic organisms. Relaxed selection after duplication along with inherent functional constraints are thought to determine the fate of the paralogs and, ultimately, the evolution of gene function. Here, we investigated the rate of protein evolution (as measured by dN/dS ratios) before and after the WGD in the hemiascomycete yeasts, and the way in which changes in such rates relate to molecular and biological function.

**Results:**

For most groups of orthologous genes (81%) we observed a change in the rates of evolution after genome duplication. Genes with atypically-low dN/dS ratio before the WGD were prone to increase their rates of evolution after duplication. Importantly, the paralogs were often different in their rates of evolution after the WGD (50% cases), however, this was more consistent with an asymmetric deceleration in the protein-evolution rates, rather than an asymmetric increase of the initial rates. Functional-category analysis showed that regulatory proteins such as protein kinases and transcription factors were enriched in genes that increase their rates of evolution after the WGD. While changes in the rate of protein-sequence evolution were associated to protein abundance, content of disordered regions, and contribution to fitness, these features were an attribute of specific functional classes.

**Conclusions:**

Our results indicate that strong purifying selection in ancestral pre-duplication sequences is a strong predictor of increased rates after the duplication in yeasts and that asymmetry in evolution rate is established during the deceleration phase. In addition, changes in the rates at which paralogous sequences evolve before and after WGD are different for specific protein functions; increased rates of protein evolution after duplication occur preferentially in specific protein functions.

**Electronic supplementary material:**

The online version of this article (doi:10.1186/s12862-017-0895-1) contains supplementary material, which is available to authorized users.

## Background

Analyses of genomic sequences indicate that whole-genome duplication (WGD) events have had strong influence in the genomes and evolution of several eukaryotic lineages of vertebrates [1], flowering plants [2], and fungi [3, 4]. Compared to duplication events that occur at smaller scales, WGD increases the potential of functional and morphological innovation by increasing the rate of duplicate retention of specific types of genes [5]. In particular, the retention of dosage-sensitive paralogs can be favored, leading to regulators and subunits of protein complexes in more than one copy [6, 7]. Likewise, regulatory and developmental genes have evolved by WGD. It has been documented that genome duplications are responsible for 90% of the increase in regulatory genes in *Arabidopsis thaliana* [8, 9]. In vertebrates, homeotic genes and multiple receptors have expanded preferentially by means of WGD [10, 11]. Therefore, the great morphological complexity and diversity in eukaryotes is, to a large extent, an effect of the amplification of the regulatory repertoire by genome duplication.

The budding yeast *Saccharomyces cerevisiae* evolved from an ancestor which underwent a WGD event, dated ~100 mya [3, 4]. This genome duplication in the hemiascomycete yeasts is perhaps the best documented event of its kind. Recent phylogenetic analysis strongly suggest that the baker's yeast genome is the result of an interspecies hybridization followed by WGD to restore fertility [12, 13]. Almost 10% of the genes were retained in two copies after the genome duplication, giving rise to more than 500 extant paralogous gene-pairs [14]. Even though only a small proportion of genes were retained in duplicate, the WGD had an impact on the lifestyle and metabolism of yeast species. For instance, it has been suggested that gene duplication improved the glycolytic flux in *S. cerevisiae*, which may have led to the colonization of high-sugar content ecological niches [15, 16].

There are biological functions associated to genes that are more likely to be retained in two copies after gene or genome duplication. It has been documented that the WGD in in *S. cerevisiae* favored the retention of ribosomal proteins, enzymes of carbohydrate metabolism, and signal-transduction kinases in duplicate [17]. Interestingly, the functional classes of paralogs retained after WGD are similar across diverse taxa ranging from yeast to *Arabidopsis thaliana* and *Paramecium* spp. [18]. Recurrent gene conversion events [19] can favor the maintenance of identity between duplicated copies of genes that code for highly constrained proteins, such as ribosomal subunits and histones [20, 21]. Still, even though WGD paralogs tend to retain an important degree of functional overlap and compensate for each other's loss [22, 23], many instances of assymetry in evolutionary rates and gene loss indicate extensive neofunctionalization after the WGD in yeast [24]. Importantly, the interspecies hybridization that gave rise to the *Saccharomyces* lineage could also have had strong implications on which genes were conserved in two copies after the WGD, as sequences were not identical at the beginning [12].

The action of natural selection before and after gene duplication has been analyzed in a systematic manner for several model organisms. These studies have shown that the rates of evolution tend to increase after gene duplication due to a relaxation of purifying selection [25–28]. Gene duplication is followed by a brief period of relaxed selection that declines rapidly, but decelerated evolutionary rates rarely revert to ancestral rates [25, 28, 29]. In addition, asymmetric rates of protein evolution between the duplicates has been documented for different eukaryotes [28, 30–32]. However, it is unclear when is this pattern of protein evolution established and to what extent it holds for different functional classes of genes that are retained in two copies after a WGD event.

In this study, we asked whether the rates of evolution change at different points in the evolutionary history of a large set of paralog pairs of the hemiascomycetes fungi, paying special attention to the rates before and after the WGD. We also asked how molecular function and other gene characteristics are associated to such evolution-rate dynamics. To this end, we took advantage of the Yeast Genome Order Browser (YGOB) compendium of sets of orthologous genes, which takes into account the phylogeny of 20 Saccharomycetaceae genomes [14]. With such catalogue, we tested different evolution models with a phylogenetic analysis by maximum-likelihood (PAML) approach. For most orthogroups, the rates of evolution increased immediately after the WGD event in a symmetric manner and, in many cases, this was followed by asymmetric deceleration of the evolutionary rates. In addition, we observed that the specific patterns of evolution depended on the molecular function of the duplicate genes, regardless of gene expression, disordered content, or contribution to fitness.

## Results

### Ancestral proteins evolving at very slow rates undergo relaxation of purifying selection after the WGD

The classic model of evolution by gene duplication suggests that the co-occurrence of two paralogs that can compensate for each other's mutations leads to increased rates in sequence divergence after duplication [33, 34]. We asked to what extent this scenario is consistent with the dN/dS ratios estimated for phylogenetic lineages that diverged before or after the WGD in the hemiascomycetes. We used a maximum-likelihood approach to test the hypothesis of different average rates of evolution between non-WGD (ω_0_) and post-WGD (ω_wgd_) branches against the null hypothesis of sequences evolving at equal rates across the phylogeny (see example in Additional file 1: Figure S1). Such tests were performed for 535 orthologous groups of genes (orthogroups) from 20 yeast species [14] in which the two WGD-paralogs (ohnologs) have been maintained in *S. cerevisiae*. Such orthogroups include both ohnologous clades and their co-orthologs in non-WGD species.

**Fig. 1:**
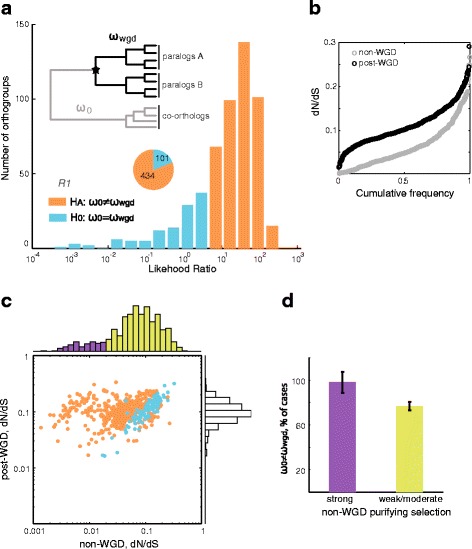
Proteins under strong purifying selection increase their rates of evolution after the WGD. **a** Frequency distribution of the Likelihood-Ratio Test (LRT) values for the hypothesis test comparing two evolutionary models. *Orange bars* indicate orthogroups in which the null hypothesis of a single dN/dS across the phylogeny is rejected in *R1* (rate-change, ω_0_ ≠ ω_wgd_); *blue bars* are orthogroups for which the null hypothesis cannot be rejected (constant-rate, ω_0_ = ω_wgd_). **b** Cumulative distribution plots comparing the average dN/dS ratios of non- and post-WGD branches. **c** Scatter plot comparing post-duplication to pre-duplication dN/dS ratios in 535 orthogroups. *Purple bars* of the *x*-axis histogram indicate orthogroups under strong purifying selection in non-WGD branches (dN/dS < 0.02) whereas lime bars are those under weak to moderate purifying selection. Each dot is a single orthogroup; *orange dots* are those in which the null hypothesis is rejected (rate-change, ω_0_ ≠ ω_wgd_), while cyan dots are orthogroups in which the null hypothesis cannot be rejected. **d** Fraction of orthogroups under strong (*purple bar*) or weak to moderate (*lime bar*) purifying selection in which the alternative hypothesis (rate-change, ω_0_ ≠ ω_wgd_) is accepted

We found that the rates of evolution are significantly different between non-WGD and post-WGD sequences for a large fraction (81%) of the analyzed orthogroups (Fig. 1a, "ω-change set", orange bars). The remaining orthogroups showed low likelihood of change in rate of evolution after WGD, thus the hypothesis of constant rates across the phylogeny could not be rejected (Fig. 1a; "ω-constant set", blue bars). The average dN/dS ratios were always less than 1.0 for both non- and post-WGD branches, but were typically higher in post-WGD sequences (*p* < 10^−60^, Wilcoxon rank-sum test), indicating a general trend of relaxation of purifying selection after the WGD event (Fig. 1b). We repeated this analyses after removing all orthogroups corresponding to ribosomal proteins, since such genes are subject to recurrent gene-conversion events after duplication [20]. A slightly larger fraction (86%) of 480 such orthogroups showed a change in evolutionary rate after duplication, and the median dN/dS ratio was also significantly higher in post-WGD branches (*p* < 10^−71^), indicating that ribosomal-protein genes were not a major driver of the observed trends. Together, these results confirm that most WGD genes increase their dN/dS ratio after duplication.

To further describe the changes in the rates of evolution, we compared the average dN/dS ratios of non-WGD and post-WGD sequences within each orthogroup. A vast majority of orthogroups fell in or above the diagonal; only a minor fraction showed the opposite scenario in which the rates of evolution decreased after duplication (Fig. 1c). We found that the overall distributions of evolutionary rates were different for branches before and after the WGD, with post-duplication sequences showing a more uniform distribution of rates centered at around dN/dS = 0.1; non-WGD sequences showed a wider distribution of their evolutionary rates. Importantly, we observed that yeast orthogroups could be readily divided in two groups based on their non-WGD dN/dS rates (Fig. 1c, yellow/purple histogram bars). The null hypothesis of equal evolution rates was more likely to be rejected in the orthogroups with very low non-WGD dN/dS rates (*p* < 10^−8^, Fisher's exact test); the difference was also significant after excluding ribosomal-protein orthogroups (*p* < 10^−5^). Indeed, virtually all orthogroups (98%) under such strong purifying selection before duplication changed their rates of evolution after the WGD event in the Saccharomycetaceae family (Fig. 1d).

To test for sensitivity in sampling, we carried out our analysis using a different data set of groups of orthologous genes in yeasts, namely from the Fungal Orthogroups Repository (FOR) [35]. This data set includes sequences from species which diverged from each other much before the WGD in the baker's yeast lineage, such as those from the *Candida*, *Yarrowia*, *Aspergillus*, *Neurospora*, and *Schizosaccharomyces* genera. A high likehood of change in the rates of protein evolution after duplication was obtained for most of the orthogroups that were represented in both data sets (Additional file 2: Figure S2, lime dots in panel *A*). Overall, 70.8% (274 out of 387) of the ω-change orthogroups from either data set showed a significant change in the rates of protein evolution in both analyses (Additional file 2: Figure S2, panel *B*). While the dN/dS ratios were similar for post-WGD sequences in both data bases, sequences from non-WGD sequences showed important differences (Additional file 2: Figure S2, panel *C*). This was expected given that the YGOB and FOR databases differ mostly in terms of sampling from species that diverged before the WGD.

### Many orthogroups show asymmetric evolution after duplication, which is consistent with differences in deceleration rather than in acceleration rates

**Fig. 2:**
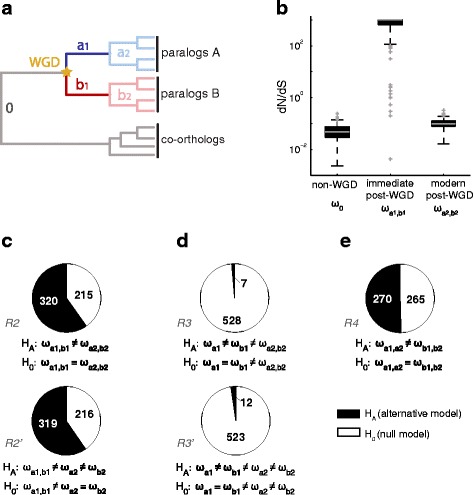
Initial post-WGD acceleration is followed by asymmetric deceleration in protein evolution rates. **a** Schematic representing the different branches used in the hypothesis testing for differences in the rates of protein evolution at different branches in the genes' phylogeny. Branches 0 (*gray*) indicate sequences from all species that diverged before the WGD (*yellow star*); branches a_1_ (*blue*) and b_1_ (*red*) are the two branches following immediately after the WGD, while a_2_ (*light blue*) and b_2_ (*pink*) are the modern post-WGD paralogous clades. **b** *Box* plots showing the distributions of dN/dS rates at different stages of the phylogeny of orthogroups for which the *R2* hypothesis (ω_0_ ≠ ω_a1,b1_ ≠ ω_a2,b2_) was accepted. Pie charts show the number of orthogroups in which the alternative hypothesis of different evolutionary rates is accepted for **c** immediate post-WGD and modern post-WGD branches (*R2* and *R2'* variant), **d** immediate post-WGD paralogous branches (*R3* and *R3'* variant), and **e** entire post-WGD paralogous clades (*R4*)

To examine whether the rates of paralogous-protein evolution have changed at different points of the yeasts phylogeny, we generated a set of new hypothesis tests (Fig. 2a-e). First, we confirmed that the WGD was followed by a brief period of accelerated protein-evolution [25, 28, 29]. Specifically, the median dN/dS rate of immediate post-WGD was higher than that of ancestral branches (*p* < 10^−108^, Wilcoxon rank-sum test), while modern post-WGD had a lower median rate than that of immediate post-WGD branches, but still higher than the typical non-WGD rate (*p* < 10^−49^) (Fig. 2b). In terms of individual cases of orthologous proteins, we found that 320 (59.8%) orthogroups were consistent with a scenario of different non-WGD, immediate post-WGD, and modern post-WGD branches (ω_0_ ≠ ω_a1,b1_ ≠ ω_a2,b2_; Fig. 2c), which again confirms that increased rates of evolution immediately after WGD are in many cases followed by a deceleration phase.

Paralogs frequently evolve asymmetrically after duplication. We therefore asked whether asymmetric changes in the rates of evolution could better explain the natural history of fungal orthogroups. To this end, we first tested the hypothesis of different acceleration rates, namely different evolutionary rates between the paralogous in immediate post-WGD branches. Intriguingly, the null hypothesis of equal rates between the two paralogous branches (ω_a1_ = ω_b1_) could not be rejected in virtually any of the orthogroups (Fig. 2d). In contrast, the null hypothesis of equal rates between the entire post-WGD clade (ω_a1,a2_ = ω _b1,b2_) was rejected for a considerable fraction (50.5%) of cases (Fig. 2e), consistent with previous observations of asymmetric evolution of paralogs. These results suggest that asymmetric evolution is established during the deceleration phase (return of constraint), rather than during the initial stage of increase in protein-evolution rate (relaxation of constraint).

To ask whether asymmetric rates were linked to strong purifying selection in non-WGD branches, we compared the proportion of cases in which the paralogous clades showed significantly-different rates, both for orthogroups with strong non-WGD purifying selection and those with weak-to-moderate purifying selection (as previously defined in Fig. 1b, c). We found no significant difference in the numbers of genes in such classes (*p* = 0.61, Fisher's exact test), indicating that a strong purifying selection before duplication is a good predictor of rate-change after duplication, but not of whether such changes are symmetric or asymmetric.

### Different protein functions show contrasting patterns of sequence evolution

**Fig. 3:**
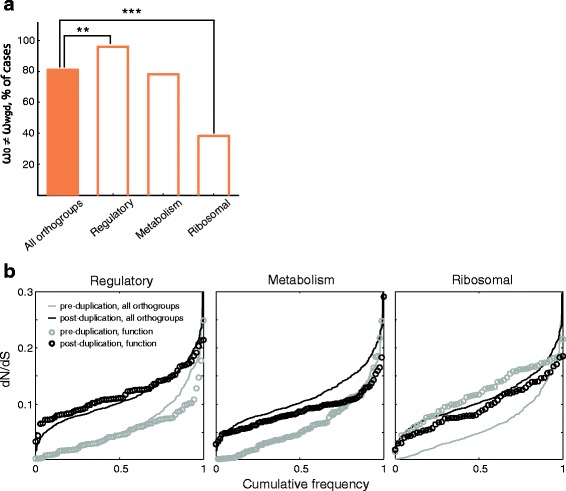
Different protein functions show different patterns of evolution before and after WGD. **a** Fraction of orthogroups for which the alternative hypothesis (ω_0_ ≠ ω_wgd_) is accepted; plot shows data for all orthogroups and different functional categories. Functional categories which are enriched or depleted in ω-change orthogroups are shown (**p < 0.01 and ***p < 0.001, *χ* ^2^ test). **b** Cumulative distribution plots of the dN/dS ratios for three different functional categories. Gray lines (all genes) and circles (specific function) show the pre-duplication dN/dS ratios; black lines and circles indicate post-duplication ratios

Following the WGD, many cytosolic ribosomal proteins, enzymes (specially carbohydrate metabolism), and regulatory proteins (mostly kinases) were retained in two copies in *S. cerevisiae* [17]. Indeed, ~39% of the orthogroups in our analysis are related to such three protein functions (71 regulatory proteins, 82 enzymes, and 55 ribosomal proteins). Using this set of highly-retained duplicates, we asked whether proteins with different biochemical roles display similar or disparate patterns of rate-change dynamics. Our results showed that the fraction of ω-change orthogroups depended on protein function. For instance, duplicated regulatory genes (protein kinases and phosphatases and transcription factors) were enriched in ω-change orthogroups, while ribosomal proteins were depleted in such scenario (Fig. 3a). Regulators were also enriched in ω-change orthogroups after removing all ribosomal-protein genes from the analysis (*p* = 0.001). Metabolic enzymes showed similar trends of changes in evolution rate as the complete set of orthogroups; the same pattern was observed when focusing only on 32 enzymes with carbohydrate-metabolism role (data not shown).

While a general increase of the typical dN/dS ratio was observed after duplication in the "all orthogroups" category (see Fig. 2b), regulatory proteins such as protein kinases were a special case in which this trend was exacerbated; median non-WGD and post-WGD dN/dS ratios were significantly different between each other (ω_0_ ≠ ω_a,b_; Fig. 3b; *p* < 10^−15^, Wilcoxon rank-sum test). The median ratios were also different in the cases of metabolic enzymes (*p* < 10^−5^) and ribosomal proteins (*p* = 0.007). In fact, the typical rates of regulatory proteins were marginally higher than all orthogroups only in post-duplication branches, while this was not the case for metabolic enzymes (black dots and black lines in Fig. 3b). Interestingly, ribosomal proteins evolved much faster than other genes in nonWGD branches, but appeared to evolve slower after gene duplication. This is most likely do to recurrent gene conversion events that have taken place in this set of highly constrained genes [20]. Taken together, these results suggest that biochemical function determines the patterns of sequence evolution of genes retained after long evolutionary times following WGD. In particular, the acceleration of protein-evolution rate is specific to certain biological functions, such as regulatory proteins.

### Protein abundance, disorder content, and contribution to fitness are determinants of the change in rate of evolution after WGD, but are strongly associated to function

**Fig. 4:**
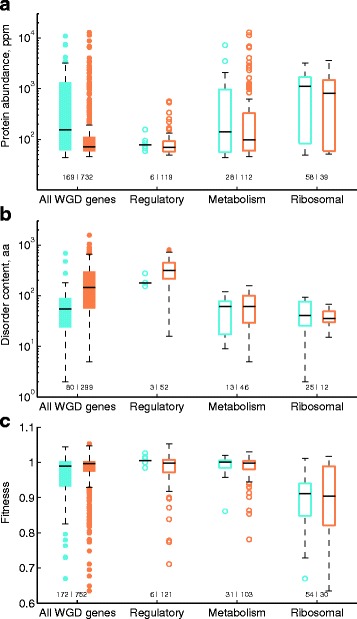
Protein function influences the association of abundance, disordered content, and contribution to fitness with the change in rate of evolution after the WGD. *Box plots* show *S. cerevisiae* data of **a** absolute protein abundance (ppm), **b** intrinsic disorder content (number of amino acids), and **c** relative fitness of the single-gene deletion for all orthogroups and genes with specific functional annotations. *Blue and orange box* plots indicate ω-constant and ω-change (ω_0_ ≠ ω_wgd_) orthogroups, respectively. Numbers below each *box plot* indicate the corresponding sample size

The rate of coding-sequence evolution depends on the level of functional constraint of the protein. In particular, gene expression is an important determinant of the rate of protein evolution, whereby highly abundant proteins evolve at slower rates [36, 37]. Selection against errors in translation, protein folding, or protein misinteraction are thought to explain this trend [38]. We therefore asked to what extent protein abundance could explain the changes in the rates of evolution after duplication and how it relates to protein function. To this end, we used quantitative data of absolute GFP-tagged protein abundances in *S. cerevisiae* [39]. We compared the distributions of protein abundances between individual paralogs in ω-change and ω-constant orthogroups (Fig. 4a). In agreement with previous inferences, we confirmed that the ω-constant proteins are typically more abundant in *S. cerevisiae* than the ω-change paralogs (*p* < 10^−10^, Wilcoxon rank-sum test, median protein abundance levels were 155 and 70.9 ppm for ω-constant and ω-change paralogs, respectively). Different protein functions were associated to important differences in protein abundance, as expected (*e.g.* ribosomal proteins were more abundantly expressed than regulatory proteins). However, the general trend of higher protein abundance of the ω-constant versus the ω-change set was not evident and not significant within different protein functions (Fig. 4a; *p* = 0.43, *p* = 0.65, and *p* = 0.21 for regulators, enzymes, and ribosomal proteins, respectively). It must be noted, however, that the small sample size in certain cases of functional groups may account for the lack of statistical significance in the differences of protein abundance between the ω-constant and ω-change sets.

To gain further insight on the functional constraints associated to the change in evolutionary rates after duplication, we also considered the relation between changes in rate of evolution and content of disordered amino acids. While many proteins need to adopt a well-defined structure to carry out their function, a large fraction of the proteome consists of polypeptide segments that are not likely to form a defined three-dimensional structure, but are nevertheless functional [40]. Such disordered proteins have been observed to evolve at high rates [41]. Indeed, we confirmed that the ω-change set of WGD paralogs was associated to *S. cerevisiae* proteins with a higher content of disordered amino acids, compared to the ω-constant set (Fig. 4b; *p* < 10^−11^, Wilcoxon rank-sum test). The median number of amino acids in disordered regions was 55 and 144 for ω-constant and ω-change proteins, respectively. However, when looking at individual functions, no difference was observed in the median content of disordered amino acids of the ω-constant and ω-change gene sets (*p* = 0.13, *p* = 0.38, and *p* = 0.46 for regulators, enzymes and ribosomal proteins, respectively). Important differences were observed among functional groups in terms of disorder content: while regulatory proteins had a significantly higher median content of amino acids in disordered regions than the entire set of orthogroups (*p* < 10^−10^), both metabolic enzymes and ribosomal proteins had typically a lower content (*p* < 10^−5^ and *p* < 10^−7^, respectively). Thus, the number of amino acids in disordered regions was more a property of the functional category and was not directly associated with evolution-rate changes after the WGD.

Essential and non-dispensable genes typically evolve at slower rates than dispensable genes [36, 42]. However, gene duplication may hide the fitness cost of single-gene deletions due to the functional compensation provided by the functionally-overlapping paralogous gene. Indeed, the fitness cost of deleting single WGD genes is usually small [22]. We took advantage of available genome-wide high-resolution fitness data of single-gene knockouts in *S. cerevisiae*. Consistent with previous observations, we found that ω-constant orthogroups had individual paralogs with a higher contribution to fitness (lower fitness of gene deletion) than paralogs in the ω-change set (Fig. 4c; *p* < 10^−3^, Wilcoxon rank-sum test). However, once again this depended more on the functional group; no difference was observed within each of the functional groups (*p* = 0.11, *p* = 0.51, and *p* = 0.90 for regulators, enzymes and ribosomal proteins, respectively). As expected, ribosomal proteins usually had a strong contribution to fitness in *S. cerevisiae* and maintained a constant evolutionary rate in non-WGD and post-WGD branches of the phylogeny. Actually, we observed no significant difference between ω-constant and ω-change orthogroups after removing all ribosomal proteins from the analysis (*p* = 0.39), indicating that the association between rate-change and fitness contribution is driven primarily by ribosomal-protein genes, which have a strong contribution to fitness and tend to show no change in rate due to gene conversion. Together, these results suggest that an interplay between the inherent gene-function 'dispensability' and the degree of functional compensation and divergence between each paralog pair determines the relationship between fitness cost of gene deletion and change in rate of evolution after WGD.

Together, these results confirm that changes in the rate of protein sequence evolution after WGD are associated to protein features such as abundance, content of disordered regions, or contribution to fitness, but are strongly connected to protein function.

### Functional analysis of duplicate pairs with asymmetric evolution

**Fig. 5:**
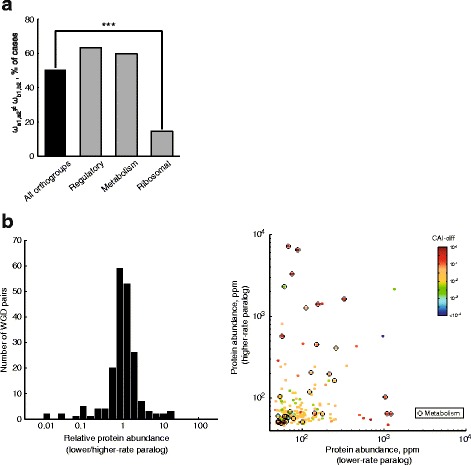
Functional analysis of asymmetric changes in the rates of evolution after WGD. **a** Fraction of orthogroups for which the alternative *R4* hypothesis (ω_a1,a2_ ≠ ω_b1,b2_) is accepted; *plot* shows data for all orthogroups (*black bar*) and different functional annotations (*gray bars*). Functional annotations depleted in asymmetric-rate orthogroups are shown (***p < 0.001, *χ* ^2^ test). **b** Histogram shows the distribution of protein abundances of the low-rate paralog relative to its high-rate paralog. **c** Scatter plot shows absolute protein abundances of the high-rate paralog compared to its low-rate paralog. *Color* scale indicates the absolute difference between the Codon Adaptation Indexes of the paralogs (CAI-diff). *Black circles* indicate all genes encoding for metabolic enzymes

Finally, we asked whether asymmetric paralogous evolution was more common in certain protein functions. We observed that regulators and metabolic enzymes had a higher fraction of paralogs with asymmetric rates of evolution, but these differences were not significant (Fig. 5a). Ribosomal proteins were significantly depleted in such scenarios of asymmetric rates after the WGD, as expected given their structural and functional constraints and, more importantly, recurrent gene-conversion events that have taken place in this set of genes after duplication.

Differences in protein abundance between the paralogs may explain asymmetry in the rates of evolution. To test this idea, we used data from *S. cerevisiae* to compare the abundances of proteins encoded by the slow and fast-evolving paralogs (only within the 270 orthogroups that showed asymmetric rates of evolution between the two paralogous clades in test *R4*). While some of the slow-evolving paralogs were relatively more abundant, we observed that the distribution of relative expression levels was mostly centered around one (*i.e.* both paralogs were expressed at similar levels) and that in some cases the paralog from the high-rate clade was actually present at higher relative levels (Fig. 5b, c).

Focusing on different functional annotations, we found that metabolic enzymes were not consistent with the expected trend: only in three cases the more abundant *S. cerevisiae* paralogous protein belonged to the low-rate clade (stars in Fig. 5c). Most of this trend could be explained by strong differences in codon usage bias, suggesting that expression change followed by codon-usage change may be the dominant force acting on several genes encoding for metabolic enzymes. Meanwhile, regulatory proteins were usually expressed at low levels in both paralogs (data not shown); where some difference in relative-protein abundance was observed, the bias was either towards the low or fast-rate paralog. Hence, differences in expression were not enough to explain the asymmetric rates within protein kinases and transcription factors. In conclusion, the fact that highly-abundant proteins evolve at lower rates than their less-abundant WGD paralogs holds only for specific cases of duplicate genes.

## Discussion

We examined the changes in evolutionary rates of duplicate-gene sequences after the WGD in the hemiascomycetes yeasts, using a maximum-likehood approach within individual groups of orthologous genes. We took advantage of the highly curated set of WGD paralogs provided by the Yeast Genome Order Browser (YGOB) project [14]. In doing so, our study expands on previous work by Scannell and Wolfe [28], who used amino-acid evolution models on concatenated sequences from eight yeast species to describe the changes protein sequence evolution and asymmetric evolution following WGD in the baker's yeast lineage. Moreover, our approach allows to interrogate the protein-evolution dynamics at different points in the natural history and for different types of proteins, providing further insight on the impact of WGD in the evolution of gene function.

Our results indicated that a significant increase in the dN/dS ratio occurred after the WGD in most orthogroups in which both paralogs were maintained. This trend of accelerated protein evolution after gene duplication has been consistently observed in yeast and other eukaryotes in different groups of paralogs and their co-orthologues [25–28]. Interestingly, we identified a group of proteins under strong purifying selection, which in almost all cases increased their rates of evolution after duplication. This, together with the fact that conserved proteins are more likely to be retained in duplicate [27], suggests that the WGD had a global effect on the proteome through a general relaxation of purifying selection in a considerable set of highly conserved proteins.

**Fig. 6:**
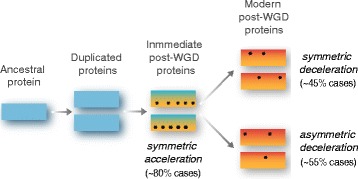
Stages of protein evolution after WGD. Immediately after duplication both paralogous copies undergo an initial phase of accelerated rate of evolution that affects the two gene copies in a similar manner (symmetric loss of evolutionary constraint), followed by a phase of deceleration in the rates of evolution (return to constraint). The degree of deceleration can be similar for both copies (symmetric return to constraint) or different between the paralogous copies (asymmetric return to constraint). *Asterisks* illustrate non-synonymous substitutions in the genes' sequences

More than 50% of the orthogroups showed asymmetric rates of protein evolution between the two paralogs, consistent with previous inferences for yeast and other eukaryotes [28, 30–32]. However, our detailed evolutionary analysis in which we interrogated the evolutionary rates at different points of the phylogeny suggested that overall rate asymmetry is established during the modern post-WGD deceleration phase (return to constraint), rather than in the initial phase of relaxation of constraint following the WGD (see model in Fig. 6). In terms of duplicated-gene function, such an asymmetric deceleration of protein-evolution rate is consistent with a subneofunctionalization scenario in which rapid subfunctionalization during the initial relaxation of purifying selection is followed by prolonged neofunctionalization during the asymmetric-deceleration phase [43].

In addition, we found that proteins with different functions can show contrasting patterns of sequence evolution. Protein kinases, transcription factors, and other regulatory proteins were very likely to change their rates of sequence evolution after the WGD. Accordingly, accelerated evolution rates in kinases and regulatory proteins has been previously inferred by comparing *S. cerevisiae* paralogs with their non-WGD *Kluyveromyces waltii* co-orthologs [4]. In addition, there is recent evidence of signal-transduction pathway expansion by means of genome duplication in *Phycomyces blakesleeanus* and other Mucorales fungi [44]. These observations suggest that signal-transduction pathways may have experienced important functional changes after the WGD, given that many protein kinases evolved slowly in ancestral branches and increased significantly after duplication. For example, the *S. cerevisiae* paralogs *DBF2* and *DBF20*, and their *Candida albicans* co-orthologue *CaDBF2* belong to an orthogroup with a strong and significant change in evolutionary rates after duplication (Additional file 3: Table S1). The essential *CaDBF2* gene from *C. albicans* codes for a single protein with a role in cytokinesis and mitotic spindle organization [45]. In *S. cerevisiae*, Dbf2 and Dbf20 are serine/threonine kinases with a role in cytokinesis [46]. While these paralogs have partially overlapping roles, there is experimental evidence of functional divergence: *DBF2* is expressed at a higher level that its paralog and also has a more prominent role in mitosis compared to Dbf20 [46, 47]. Interestingly, Dbf2 has also been shown to play a role in *S. cerevisiae* regulating the stability of mRNAs by co-transcriptional binding [48].

Transcription factors were among the proteins that were more often subject to strong purifying selection before the WGD in yeasts (Fig. 3). Gene duplication plays an important role in the evolution and functional diversification of transcription factors; the great morphological complexity in plants and animals could be a consequence of the amplification of the regulatory repertoire by WGD [10, 11]. Changes in the amino acid sequences of such regulatory proteins were limited by their functions and that such strong constraint was relieved after gene duplication. In a genome duplication context, this could have had important implications in the systems-level evolution of regulatory pathways. Indeed, it has been shown that WGD lead to widespread partitioning of expression networks in yeast [49].

Our results showed that genes with significant changes in the rates of protein evolution after WGD share some features. Proteins in ω-change orthogroups were less abundant, had higher content of intrinsic disorder, and were more dispensable in *S. cerevisiae* than ω-constant orthogroups (Fig. 4). However, such features were intrinsically associated to molecular function, rather than to changes in the rates of evolution after the WGD. For instance, orthogroups of ribosomal proteins are highly expressed and have a stronger contribution to fitness, while protein kinases and other regulators tend to have higher intrinsic-disorder content. Indeed, it has been suggested that protein function sets important constraints in the way in which protein sequences change with time [42]. It must be noted that an important limitation is that most data has been collected in the budding yeast *S. cerevisiae* and less experimental data is available across other yeasts across the phylogeny. It would be interesting to analyze, for instance, whether proteins in orthogroups with atypical slow rates before WGD, such as kinases, are more abundant--and thus evolved slower--in species that diverged from *S. cerevisiae* before the WGD. Asymmetric evolution was rarely observed for ribosomal proteins, which were clearly depleted in orthogroups in which the evolution rates were significantly different among pre-duplication and both post-duplication branches. This could be readily explained by recurrent gene-conversion events after duplication in WGD paralogs coding for cytosolic ribosomal proteins [20].

Finally, we found that differences in expression level, based on available data for *S. cerevisiae*, were not always associated to the asymmetric rate of evolution. We did find both cases of orthogroups in which the more abundant protein evolved either at a significantly slower or faster rate compared its less abundant paralog. Among the most evident cases of the latter were the Cdc19 and Pyk2 pyruvate kinases, which catalyze the conversion of phosphoenolpyruvate to pyruvate in the final step of glycolysis. *CDC19* is essential for growth on glucose or other fermentable sugars as the sole carbon source, but are dispensable for growth on ethanol or lactate, indicating that there is an alternate route for pyruvate synthesis [50]. Overexpression of *PYK2* restores growth on glucose in *cdc19* mutant cells [51]. High Cdc19 levels are due to activation by fructose-1,6-bisphosphate (FBP), while the *PYK2* paralog is subject to glucose repression and appears to be insensitive to FBP levels, suggesting that it is active when FBP levels are too low to activate *CDC19* [51]. The phosphogluconate dehydrogenase isoenzymes are a similar instance of the slower-evolving paralog expressed at a higher level [52]. *GND1* is the major isofrom, is induced during growth on D-glucono-delta-lactone, and is repressed during growth on ethanol or lactic acid [52]. In both *CDC19/PYK2* and *GND1/GND2* cases, enzymes of the paralogous pairs have similar biochemical roles, but are expressed in contrasting patterns and display strong differences in their codon-adaptation biases. This was also the case for other metabolic-gene pairs with strong evolutionary-rate and expression asymmetry, namely *GDH1/GDH3*, *GPD1/GPD2*, *TRR1/TRR2*, and *ADE16/ADE17*. Changes in gene expression followed by changes in codon usage are likely the dominant evolutionary force acting on several metabolic genes.

## Conclusions

Our results indicate that the most prominent cases of changes in protein evolution rate after the WGD in yeast occurred in proteins that were subject to strong purifying selection before duplication, which experienced a brief increase in protein-sequence evolution [28] followed by an asymmetric deceleration in their rates of evolution. An asymmetric return to evolutionary constraint could lead to important functional differences among proteins from species that diverged after the WGD; characterizing the orthologues of gene duplicates or reconstructing ancestral proteins [53] will provide valuable information to understand the connection between sequence and functional divergence of duplicate genes. In addition, we found that the way in which protein-evolution rates changed after the WGD in yeasts is different for different protein functions. Importantly, it remains to be analyzed to what extent and how the inter-species hybridization event that likely preceded the WGD in yeasts [12] may have impacted the evolutionary-rate dynamics and functions of WGD paralogs; many paralogous branches may have actually began as non-identical orthologues. It can be anticipated that focusing on specific functional groups across the yeasts phylogeny will provide valuable information to understand the patterns of evolution of paralogous proteins in the context of genome duplication.

## Methods

### Acquisition of sequences, alignments, and phylogenetic trees

We obtained the protein amino acid and DNA sequences from the Yeast Genome Order Browser (YGOB, http://ygob.ucd.ie/), which contains sequences from 20 hemiascomycete species: twelve species diverged from one another after the WGD and eight diverged before the WGD [14]. Distantly-related species such as *Candida albicans* and *Schizosaccharomyces pombe* are excluded from the database due to low gene-order conservation. The YGOB database is a manually curated database that uses both sequence similarity and genome context (synteny) to assess the homology relationships of genes. It provides groups of ohnologs and their co-orthologs (pillars) from all species; here we refer to such pillars as orthogroups (groups of orthologs). To our ends, we selected orthogroups which contain the two WGD paralogous clades with the two gene copies retained at least in *S. cerevisiae* (ohnologs) and their pre-duplication co-orthologues from non-WGD species in the phylogeny (see example in Additional file 1: Figure S1).

Starting from 546 orthogroups, groups with less than 13 sequences and with less than three nonWGD sequences were discarded; the remaining 535 orthogroups were used for further analysis. Individual files containing the DNA and amino acid sequences from each orthogroup were organized and the stop codons from DNA sequences were trimmed and compared with correspondent amino acid sequences. The amino acid sequences of each orthogroup were aligned with MUSCLE 3.6 [54]. Nucleotide alignments with PAML format were generated using the amino-acid alignments as a template with PAL2NAL [55]. Finally, the phylogenetic trees were generated based on the topology of the species phylogeny retrieved from the YGOB database and adapted to each orthogroup using the ETE3 toolkit, which allows the manipulation of tree topology by rooting, pruning, or sorting branches [56].

In addition, we used sequences from an alternative data set, the Fungal Orthogroups Repository (http://www.broadinstitute.org/regev/orthogroups/), which contains sequences from 23 ascomycete species. In this case, the data set includes distant related species such as *S. pombe* [35]. This non-supervised database was generated using the SYNERGY algorithm [57], which uses sequence similarity and a given species phylogeny to reconstruct the evolutionary history of all genes in a large group of species. As a result, gene trees that reconstruct the evolutionary history of the genes, including gene duplications and losses, are obtained. Here, each gene orthogroup contains all of the orthologous genes present in species of the database [35]. Again, we selected orthogroups which contain the two WGD paralogous clades with the two gene copies retained at least in *S. cerevisiae*. From 545 orthogroups, we removed 83 based on sequence quality (frequency of undetermined nucleotides) and quantity (at least six gene sequences), and took the remaining 462 orthogroups for further analysis. We generated the alignments as described above for sequences from the YGOB database.

### Phylogenetic analysis by maximum likelihood with CodeML

Once we had all the curated alignments and the corresponding phylogenetic trees, we contrasted in each case two evolutionary models using CodeML, a package from PAML 4.4 [58]. To test for differences in the evolution rates before and after WGD, in a first hypothesis test we identified the WGD in each phylogeny based on the topology of the tree and grouped the branches in non-WGD (ω_0_) and post-WGD (ω_wgd_) sequences. Then, we used these topologies, alignments, and trees as CodeML input using a standard control script. As an alternative model (*R1*), we used the user-specified “branch” model which allows the calculation of a distinct ratio for the ω_0_ and ω_wgd_ branches (Codonfreq = 2). This alternative model was compared with the null model in which all the branches have the same dN/dS ratio. The likelihood ratio test was used to compare the null and alternative models and was calculated as 2(*L*_1_–*L*_0_), where *L*_1_ is the maximum likelihood value of the alternative hypothesis and *L*_0_ the maximum likelihood value of the null hypothesis. A *χ* ^2^ distribution with one degree of freedom was used to calculate the *p*-value; null hypotheses were rejected with a *p* < 0.05 cutoff (see example in Additional file 1: Figure S1). Using this same approach, we generated different pair of null-alternative hypotheses (*R2*, *R3*, and *R4*, see Fig. 2) to test for differences in the rates of evolution at different points in each of the orthogroups.

### Protein functional groups, abundance, fitness, and intrinsic disorder data

Functional categories were manually defined based on the functional annotations for the *S. cerevisae* paralogs obtained from the Saccharomyces Genome Database (http://www.yeastgenome.org). The regulatory function includes genes annotated as protein kinases, phosphatases, transcription factors, and proteins involved in chromatin remodeling. Metabolic function included enzymes involved on biosynthetic and catabolic processes, while ribosomal function included proteins which are structural components of the ribosome. In addition, we used the protein abundance data based on spectral counting of strains expressing GFP-tagged proteins [39]. Fitness data was obtained from high-resolution competition-based experiments describing the fitness of single-gene deletions relative to wild-type cells [59]. Finally, intrinsic disorder content data was from Landry et al. [60], where intrinsically unstructured regions were predicted using the DISOPRED algorithm [61].

Additional file 3: Table S1 includes all dN/dS data, likelihood-ratio tests' results, and *S. cerevisiae* protein properties and functions for the 535 orthogroups of the YGOB database, while Additional file 4: Table S2 includes results for 462 orthogroups using the FOR database.

## Additional files

Additional file 1: Figure S1. Analysis of evolution rates in fungal WGD orthogroups. The *S. cerevisiae PSK1/PSK2* orthogroup is shown as an example of significantly relaxed purifying selection after duplication. Gray branches indicate the orthologs of *PSK1/PSK2* in ascomycetes species that diverged before the WGD (yellow star), while black branches indicate post-WGD species with two paralogous clades. Average dN/dS ratios are shown for both non-WGD (ω_0_) and post-WGD genes (ω_wgd_); the *p*-value is from the likelihood ratio test of the alternative hypothesis of different rates of protein evolution between ω_0_ and ω_wgd_. (EPS 1264 kb)
Additional file 2: Figure S2. Results are consistent for most orthogroups when using two alternative data sets. (A) Comparison of Likelihood-Ratio Test (LRT) values for the hypothesis test comparing the two evolutionary models (null: ω_0_ = ω_wgd_; alternative: ω_0_ ≠ ω_wgd_) using sequences from two different datasets: the Yeast Genome Order Browser (YGOB) [14] and the Fungal Orthogroups Repository (FOR) [35]. (B) Venn diagram of orthogroups in which the alternative hypothesis (ω_0_ ≠ ω_wgd_) is accepted in the YGOB and the FOR data sets, (C) Comparison of non-WGD (gray dots) and post-WGD dN/dS ratios of 426 orthogroups calculated for sequences for the YGOB and the FOR data sets. (EPS 2241 kb)
Additional file 3: Table S1. YGOB data set analysis, including dN/dS data, likelihood-ratio tests' results, and *S. cerevisiae* protein properties and functions for 535 orthogroups. (XLSX 242 kb)
Additional file 4: Table S2. FOR data set analysis, including dN/dS data, likelihood-ratio tests' results for 462 orthogroups. (XLSX 57 kb)

## Abbreviations

LRT: Likelihood-ratio test
PAML: Phylogenetic analysis by maximum likelihood
WGD: Whole-genome duplication
